# Simultaneous Bilateral Cochlear Implantation in a Child with Warsaw Breakage Syndrome and Normal Cochlear Morphology: A Case Report and Focused Literature Review

**DOI:** 10.3390/diagnostics16193092

**Published:** 2026-09-24

**Authors:** Pinelopi Samara, Michail Athanasopoulos, Nikolaos Markatos, Evangelia Koudoumnaki, Ioannis Athanasopoulos

**Affiliations:** 1 Children’s Oncology Hospital “Marianna V. Vardinoyannis-ELPIDA”, 115 27 Athens, Greece; 2 Department of Otology, Audiology, and Pediatric Neurotology, Pediatric Center of Athens, 151 25 Athens, Greece; m.athanasopoulos@med.uoa.gr (M.A.); markatoshearing@gmail.com (N.M.); koudoumnakievi@yahoo.gr (E.K.); dr.iathanasopoulos@gmail.com (I.A.)

**Keywords:** Warsaw Breakage Syndrome, DDX11, cochlear implantation, whole-exome sequencing, sensorineural hearing loss

## Abstract

**Background and Clinical Significance:** Warsaw Breakage Syndrome (WABS), also termed DDX11-related cohesinopathy, is an exceptionally rare autosomal recessive disorder caused by biallelic pathogenic variants in DDX11. Sensorineural hearing loss is a recurrent manifestation and has frequently been reported together with structural abnormalities of the cochlea or cochlear nerve. Published experience with cochlear implantation in WABS remains extremely limited. **Case Presentation:** A 2.5-year-old boy was referred because of absent spoken-language development and limited responses to environmental and speech sounds. Newborn hearing screening had not been performed. After treatment of bilateral middle-ear effusions, comprehensive audiological assessment showed absent transient-evoked otoacoustic emissions and cochlear microphonics, no reproducible auditory brainstem response waveforms at the maximum tested level of 90 dB nHL, and auditory steady-state response estimates in the severe-to-profound range bilaterally. Clinical examination revealed microcephaly, short stature, and global developmental delay, raising suspicion of an underlying syndromic disorder. High-resolution computed tomography demonstrated normal cochlear morphology, while magnetic resonance imaging showed bilaterally visualized cochlear nerves without aplasia or marked hypoplasia. Whole-exome sequencing identified a pathogenic frameshift variant in DDX11 in apparent homozygosity, supporting the molecular diagnosis of WABS. Simultaneous bilateral cochlear implantation was performed at 3 years of age without complications. Longitudinal auditory development, assessed with the LittlEARS Auditory Questionnaire, improved from 0/35 at activation to 10/35 at 8 months, 21/35 at 20 months, and 33/35 at 24 months of auditory age. At approximately 30 months of auditory age, structured language assessments demonstrated further receptive and expressive communication gains, although language development remained influenced by the underlying neurodevelopmental disorder. At 33 months of auditory age, functional auditory performance assessed with PEACH-GR was 90% in quiet, 80% in noise, and 85% overall, while Greek-standardized language testing demonstrated persistent language difficulties relative to chronological-age expectations. **Conclusions:** Severe-to-profound prelingual sensorineural hearing loss in WABS may occur despite normal cochlear morphology and bilaterally visualized cochlear nerves. In this patient, simultaneous bilateral cochlear implantation was technically uncomplicated. During subsequent bilateral device use, programming, rehabilitation, and maturation, quantitatively documented progressive auditory development and functional communication gains were observed. Comprehensive audiological, radiological, genetic and developmental assessment is essential for individualized implant candidacy and realistic family counseling.

## 1. Introduction

Warsaw Breakage Syndrome (WABS), increasingly referred to as DDX11-related cohesinopathy, is an exceptionally rare autosomal recessive disorder caused by biallelic pathogenic variants in *DDX11*, located on chromosome 12p11.21 [1]. *DDX11* encodes an ATP-dependent DNA helicase involved in DNA replication and repair, sister chromatid cohesion, and maintenance of genomic stability. Loss of DDX11 function results in chromosomal instability and impaired embryonic development, producing a clinically heterogeneous multisystem disorder [2,3,4,5,6].

Since its first description in 2010 [7], the recognized phenotype has expanded to include prenatal and postnatal growth restriction, severe microcephaly, developmental delay or intellectual disability, craniofacial dysmorphism, pigmentary abnormalities, and variable skeletal, cardiovascular, renal, and genitourinary manifestations [8,9,10,11]. Within this multisystem phenotype, sensorineural hearing loss is a recurrent and clinically important feature and is frequently bilateral and severe to profound [7,12,13]. In young children, early auditory deprivation may further compromise speech, language, cognitive, and social-communication development, particularly when a neurodevelopmental disorder is already present. The otologic phenotype of WABS is heterogeneous. Reported temporal-bone abnormalities include cochlear hypoplasia, incomplete partition, vestibular and semicircular canal malformations, internal auditory canal abnormalities and cochlear nerve deficiency [7,10,11,12,13,14,15,16]. These findings are clinically relevant because cochlear geometry and cochlear nerve integrity may influence implant candidacy, electrode-array selection, surgical planning, and expected postoperative performance. Conversely, the extent to which severe hearing loss can occur in WABS despite apparently normal inner-ear anatomy remains poorly defined.

This distinction is important in clinical practice. Conventional computed tomography (CT) and magnetic resonance imaging (MRI) characterize gross inner-ear anatomy and cochlear nerve morphology but cannot directly assess microscopic sensory structures or cochlear function. Accordingly, a child with syndromic features, severe hearing loss, and normal temporal-bone imaging still requires a comprehensive functional and etiological evaluation. Next-generation sequencing has become particularly valuable in this setting because a molecular diagnosis can unify apparently unrelated findings, guide genetic counseling and multisystem surveillance, and support individualized decisions regarding hearing rehabilitation [17,18].

Published experience with cochlear implantation in WABS remains very limited, and several previously reported recipients had cochlear malformations or cochlear nerve abnormalities [11,12,14,15,16]. Consequently, the feasibility and functional outcome of cochlear implantation in WABS with normal cochlear anatomy remain insufficiently characterized. Whether simultaneous bilateral implantation can provide meaningful auditory access in this anatomical setting is therefore clinically relevant but supported by only minimal patient-level evidence.

The purpose of the present report was to describe the audiological, radiological, genetic, surgical, and longitudinal functional findings of a child with a molecularly supported diagnosis of WABS, bilateral severe-to-profound prelingual sensorineural hearing loss, normal cochlear morphology, and bilaterally visualized cochlear nerves who underwent simultaneous bilateral cochlear implantation. We also provide a focused patient-level review of previously reported cochlear implant recipients with WABS to place the present findings in context.

## 2. Case Presentation

### 2.1. Clinical History and Initial Presentation

A 2.5-year-old boy was referred to our pediatric otolaryngology center because of absent spoken-language development and limited responses to environmental sounds and spoken language. Newborn hearing screening had not been performed. Pregnancy and delivery were reported to have been uncomplicated, and the child was born at term. There was no history of neonatal intensive-care admission, congenital infection, perinatal hypoxia, meningitis, head trauma, or exposure to ototoxic medication. The family history was negative for congenital hearing loss, developmental disorders, and known genetic conditions.

### 2.2. Clinical and Developmental Assessment

Physical examination revealed microcephaly and short stature, and developmental assessment demonstrated global developmental delay. The combination of growth abnormalities, neurodevelopmental impairment, absent spoken language, and marked auditory dysfunction raised concern for an underlying syndromic disorder and prompted a multidisciplinary etiological evaluation.

### 2.3. Audiological Evaluation

At the initial assessment, bilateral middle-ear effusions were identified. Bilateral myringotomy and ventilation tube placement were subsequently performed. After restoration of middle-ear aeration, otoscopy and tympanometry confirmed adequately aerated middle ears bilaterally, and comprehensive diagnostic audiological testing was undertaken at the Department of Otology, Audiology, and Pediatric Neurotology, Pediatric Center of Athens, to investigate hearing impairment as a potential contributor to the child’s delayed speech and language development.

Middle-ear assessment was performed using the Titan platform, and transient-evoked otoacoustic emissions (TEOAEs) were assessed using the Sera platform (Interacoustics A/S, Middelfart, Denmark). TEOAEs were absent bilaterally. Diagnostic auditory brainstem response (ABR), cochlear microphonic (CM), and auditory steady-state response (ASSR) recordings were obtained using the Eclipse evoked-potential platform (Interacoustics A/S, Middelfart, Denmark) with ER-3A insert earphones. ABR recordings used click stimulation at 21.1 stimuli/s, a 30–1500 Hz recording filter, and up to 1000 sweeps. No reproducible ABR waveform was identified in either ear at 90 dB nHL, which was the highest level tested during that examination; higher levels were not tested. CM was specifically assessed using separate condensation- and rarefaction-polarity recordings, and no reproducible polarity-reversing CM was detected in either ear. ASSR testing at 0.5, 1, 2, and 4 kHz yielded estimated thresholds in the severe-to-profound range bilaterally (Figure 1). Because ASSR obtained at very high stimulus levels may be vulnerable to artifactual responses, particularly in the assessment of profound hearing loss [19], the ASSR findings were not interpreted in isolation. The final audiological classification was based on concordance among the absent TEOAEs and CMs, absent ABR responses at the highest tested level, ASSR estimates, and the child’s clinical auditory behavior.

Because neither newborn-hearing-screening results nor earlier objective audiological assessments were available, the precise onset of the hearing loss could not be established. Nevertheless, the history of absent spoken-language development and limited auditory responsiveness, together with the objective findings at 2.5 years of age, supported classification of the hearing loss as prelingual and probably congenital.

Following confirmation of bilateral severe-to-profound sensorineural hearing loss, the child was promptly referred for cochlear implant evaluation. Given the severity of the hearing loss, the absence of functional spoken language, and the importance of limiting further auditory deprivation during early development, additional avoidable delay in hearing rehabilitation was considered inappropriate.

### 2.4. Radiological Assessment

Detailed radiological assessment was undertaken both to investigate the etiology of the hearing loss and to support preoperative cochlear implant planning. High-resolution CT of the temporal bones demonstrated normally formed cochleae bilaterally, each with 2.5 turns and preserved modiolar architecture. The round and oval windows were normally formed, and there was no evidence of cochlear hypoplasia, incomplete partition, labyrinthitis ossificans, enlarged vestibular aqueduct, vestibular abnormality, or semicircular canal malformation. The internal auditory canals were of normal caliber bilaterally.

MRI of the temporal bones and internal auditory canals demonstrated bilateral cochlear nerves, symmetrically visualized and of normal diameter, without evidence of aplasia or marked hypoplasia. No mass lesion or pathological enhancement was identified within the internal auditory canals or cerebellopontine angles, and no gross structural abnormality of the brainstem was identified (Figure 2).

### 2.5. Genetic Investigation and Molecular Diagnosis

Genetic investigation was initiated because the severe hearing loss occurred in association with microcephaly, short stature, and global developmental delay. Whole-exome sequencing was performed on genomic DNA extracted from a buccal-swab sample. Target enrichment used the Twist Human Core Exome EF Multiplex Complete Kit (Twist Bioscience, South San Francisco, CA, USA), followed by sequencing on an Illumina NextSeq 500 platform (Illumina, San Diego, CA, USA).

The analytical pipeline included 21,526 genes, and bioinformatic processing was performed using validated algorithms implemented in SOPHiA DDM version 5.10.39.1 (SOPHiA GENETICS, Rolle, Switzerland). Sequence reads were aligned to the GRCh37/hg19 human reference genome. According to the laboratory report, 95.46% of the target region was covered, with a mean sequencing depth of 50×.

Whole-exome sequencing identified the DDX11 variant NM_030653.3:c.1403dup, p. (Ser469Valfs*32), corresponding to dbSNP rs778363138, in apparent homozygosity. The frameshift introduces a premature termination codon and is predicted to result in loss of DDX11 function. According to the reporting laboratory, the variant was classified as pathogenic using the American College of Medical Genetics and Genomics/Association for Molecular Pathology criteria PVS1, PM2, and PM3 [20].

Copy-number analysis derived from the next-generation sequencing data did not identify a deletion involving part or all of DDX11, reducing the likelihood that the apparent homozygosity resulted from a large deletion affecting the second allele. The diagnostic assay was based exclusively on next-generation sequencing, and no independent Sanger confirmation or other orthogonal validation was performed. Targeted parental segregation testing was recommended but was not performed; therefore, segregation of the variant and parental carrier status could not be independently confirmed. In conjunction with the characteristic clinical phenotype, these findings supported the molecular diagnosis of WABS.

### 2.6. Cochlear Implant Candidacy and Surgical Procedure

The child was evaluated by a multidisciplinary cochlear implant team comprising pediatric otologists, audiologists, a clinical geneticist, a radiologist, pediatric specialists, and speech–language and auditory-rehabilitation professionals. The bilateral severe-to-profound hearing loss, absence of functional spoken language, normal cochlear morphology, and bilateral visualization of the cochlear nerves supported cochlear implant candidacy.

The family was counseled that the associated neurodevelopmental disorder could influence both the rate and the ultimate extent of auditory, speech, and language acquisition. The considerable uncertainty in individual prognosis was also discussed because published experience with cochlear implantation in WABS remains extremely limited.

Simultaneous bilateral cochlear implantation was performed at 3 years of age. Both cochleae were accessed without anatomical difficulty, and full insertion of both electrode arrays was achieved. Intraoperative impedance telemetry was within the expected range, and recordable electrically evoked neural responses were obtained bilaterally. No intraoperative complications occurred. Postoperative imaging confirmed appropriate intracochlear positioning of both electrode arrays, and the postoperative course was uncomplicated.

### 2.7. Device Activation, Rehabilitation, and Functional Outcomes

Both cochlear implants were activated approximately 4 weeks after surgery. The child then entered an intensive auditory–verbal rehabilitation program that included regular cochlear implant mapping, audiological follow-up, and structured speech–language intervention. For longitudinal outcome reporting, auditory age was defined as the time elapsed since bilateral device activation.

Auditory development was monitored longitudinally with the LittlEARS Auditory Questionnaire (LEAQ), a 35-item caregiver questionnaire for early auditory behavior whose multilingual validation included a Greek-language sample [21]. At activation (auditory age 0 months), the total LEAQ score was 0/35. The score increased to 10/35 at 8 months of auditory age and to 21/35 at 20 months. A further marked improvement was documented at 24 months of auditory age, when the score reached 33/35. These serial scores provide a standardized quantitative caregiver-reported measure of progressive auditory development during bilateral cochlear implant use and rehabilitation.

At approximately 30 months of auditory age, receptive and expressive language abilities were further evaluated using the Clinical Evaluation of Language Fundamentals (CELF), the Derbyshire Language Scheme Rapid Screening Test, and the South Tyneside Assessment of Syntactic Structures (STASS). Because these instruments are not standardized for the Greek pediatric population in the form used, their findings were interpreted descriptively rather than converted into normative standard scores, percentiles, or language-age equivalents. Together, these structured assessments and longitudinal therapist observations documented comprehension of familiar phrases, simple questions and verbal commands without visual cues, use of common verbs and everyday vocabulary, spontaneous production of short phrases, and emerging grammatical morphology.

At the most recent follow-up, at a chronological age of 5 years and 10 months and an auditory age of 33 months, functional auditory performance in everyday listening situations was assessed using PEACH-GR, the Greek version of the Parents’ Evaluation of Aural/Oral Performance of Children, a parent-report questionnaire validated in Greek-speaking children with typical hearing [22]. The child obtained a score of 90% in quiet, 80% in noise, and an overall PEACH-GR score of 85%. Language abilities were additionally evaluated using the Greek-standardized Action Picture Test [23] and Word Finding Vocabulary Test [24]. Based on normative data for chronological age, performance on the Action Picture Test corresponded to the 10th percentile for descriptive adequacy and below the 10th percentile for grammatical adequacy. Performance on the Word Finding Vocabulary Test corresponded to the 10th percentile. Interpretation of these language findings in relation to auditory age would provide additional developmental context, as the child had experienced 2 years and 9 months of auditory input following cochlear implant activation. However, an auditory-age-based normative comparison was not possible because the child’s auditory age was below the lower age limit of 4 years for the standardized language assessments. Accordingly, the reported percentile scores are based exclusively on chronological-age norms and should be interpreted within the context of the child’s shorter duration of auditory experience. Formal postoperative aided sound-field thresholds and standardized speech-perception scores could not yet be obtained reliably because the child was not able to cooperate consistently with the required behavioral testing. This represents a limitation of the postoperative outcome assessment.

The child’s language development remained slower than would be expected in an otherwise typically developing child, consistent with the neurodevelopmental phenotype of WABS. Nevertheless, longitudinal assessment demonstrated progressive auditory and functional communication development during bilateral cochlear implant use, regular device programming, structured rehabilitation, and ongoing maturation. The PEACH-GR findings indicate substantial functional auditory performance in everyday listening situations, whereas the standardized language assessments demonstrate persistent language difficulties relative to chronological-age expectations (Table 1). These findings should be interpreted in the context of multiple contributing factors, including age at implantation, duration of auditory experience, consistent bilateral device use and programming, structured rehabilitation, maturation, and the child’s underlying neurodevelopmental disorder. The observed outcomes represent benefits in this individual patient and should not be generalized to all children with WABS.

### 2.8. Focused Literature Search

A focused literature search was performed in PubMed/MEDLINE from database inception through July 2026 using the terms (“Warsaw Breakage Syndrome” OR “DDX11-related cohesinopathy” OR “DDX11”) AND (“cochlear implant*” OR “hearing loss” OR “deafness”). Reference lists of potentially relevant publications were also screened. Titles and abstracts were reviewed to identify reports describing patients with WABS and possible cochlear implantation. Full texts of potentially eligible publications were subsequently reviewed, and reports were included when they provided identifiable patient-level information on cochlear implantation. Publications describing the same patient were consolidated at the patient level. The search identified five unique previously reported cochlear implant recipients with WABS in the publications meeting these criteria [11,12,14,15,16]. The reports by Bottega et al. and Brotto et al. describe the same implanted patient, whereas Rabin et al. reported two implanted patients.

This search was designed as a focused narrative review rather than a systematic review. Screening was not independently duplicated, PubMed/MEDLINE was the only bibliographic database searched, and formal risk-of-bias assessment and PRISMA procedures were not applied. Consequently, reports not indexed in PubMed/MEDLINE, non-English-language publications, conference abstracts, and cases in which cochlear implantation was not emphasized in the title or abstract may have been missed. Accordingly, the five previously reported cochlear implant recipients identified by this focused search should not be interpreted as an exhaustive count of all individuals with WABS who have undergone cochlear implantation.

## 3. Discussion

The clinical relevance of the present case lies in the demonstration that severe-to-profound prelingual auditory dysfunction requiring cochlear implantation can occur in WABS despite normal cochlear morphology and bilaterally visualized cochlear nerves. This is an important distinction from much of the previously published cochlear implant experience in WABS, in which cochlear or neural malformations were prominent. The case also provides longitudinal quantitative evidence of auditory development during bilateral cochlear implant use and rehabilitation, thereby addressing both anatomical and functional aspects of cochlear implant candidacy in this ultra-rare disorder.

DDX11-related cohesinopathy remains exceptionally rare. The 2025 GeneReviews update summarized 26 individuals with biallelic pathogenic variants in DDX11 [1]. Inclusion of the additional genetically confirmed patients reported by Leal et al. [15], Molnar et al. [16], and Zhang et al. [25] brings the published total to at least 29 individuals. Because several publications describe siblings, prenatal cases [26], or patients reported in more than one article, cumulative numbers should be interpreted cautiously.

Within this small body of literature, our focused review identified patient-level information on five previously reported cochlear implant recipients with WABS [11,12,14,15,16], with the present patient adding another well-characterized case to the published experience. Among the cases identified, the present report is distinguished by the explicitly documented combination of simultaneous bilateral cochlear implantation, normal cochlear morphology, and bilateral visualization of the cochlear nerves. Rabin et al. reported bilateral cochlear implantation at 2 years of age in one patient but did not state whether the procedures were simultaneous or sequential [14]. Bottega et al., with additional audiological follow-up subsequently reported by Brotto et al., described sequential bilateral implantation in a child with bilateral incomplete partition type II [11,12]. Leal et al. reported unilateral implantation without sufficient device-specific postoperative data to assess benefit reliably [15], whereas Molnar et al. described unilateral implantation in a child with severe bilateral type I cochlear hypoplasia after individualized three-dimensional surgical planning [16] (Table 2).

This limited experience also underscores the need for careful interpretation of the audiological findings. Newborn hearing screening had not been performed; so, objective information from the neonatal period was unavailable. Current early-hearing-detection principles emphasize that absent or delayed spoken-language development and limited responses to sound should prompt comprehensive audiological assessment irrespective of previous screening results [27]. In the present patient, the diagnosis did not depend on ASSR alone. High-intensity ASSR may occasionally generate artifactual responses, particularly in profoundly hearing-impaired patients [19]; therefore, the severe-to-profound ASSR estimates were interpreted in conjunction with absent TEOAEs and CMs, absent reproducible ABR responses at 90 dB nHL (the highest level tested during that examination), and the child’s clinical auditory behavior. The absence of both TEOAEs and CMs did not support the typical electrophysiological pattern of auditory neuropathy spectrum disorder, in which cochlear responses are characteristically preserved.

Radiological findings add a complementary, but distinct, dimension to this assessment. Normal clinical imaging should not be equated with normal cochlear function. Cochlear hypoplasia, incomplete partition, vestibular and semicircular canal abnormalities, and cochlear nerve abnormalities have been described in WABS [7,10,11,12,13,14,15,16], but conventional CT and MRI cannot resolve the organ of Corti, sensory hair cells, supporting cells, the stria vascularis, or other microscopic cochlear structures [28]. In the present patient, absent TEOAEs and CMs are compatible with peripheral cochlear dysfunction but do not permit precise cellular localization of the lesion. Abnormal development or degeneration of the sensory epithelium, supporting structures, or other microscopic cochlear components therefore remains a biologically plausible but strictly hypothesis-generating explanation. No patient-specific histological or functional molecular evidence is available to demonstrate hair-cell loss, and the precise mechanism of hearing loss in WABS with normal temporal-bone imaging remains unknown.

This anatomical distinction has direct surgical implications when cochlear implantation is considered. In malformed ears, reduced cochlear dimensions, cystic apical morphology, absent or abnormal modiolar architecture, and altered turns can influence the optimal electrode length, stiffness, insertion depth, and surgical approach. Three-dimensional planning tools such as OTOPLAN can estimate cochlear dimensions, cochlear duct length, and predicted angular insertion depth and may support individualized electrode-array selection [29]. In malformed cochleae, however, equations developed for normal anatomy may be less reliable; estimation of the available outer-wall length (OWL) has therefore been proposed as an alternative anatomy-based measure for selecting an array appropriate to the extent of the malformed cochlear lumen [30]. Molnar et al. illustrated the practical relevance of individualized three-dimensional planning in WABS with type I cochlear hypoplasia [16]. By contrast, the normally formed cochleae in the present patient permitted uncomplicated bilateral access and full electrode-array insertion without anatomy-driven modification of the surgical strategy.

The genetic findings further illustrate the phenotypic variability in WABS. The c.1403dup frameshift variant identified in the present patient has been reported previously in WABS. It was described in compound heterozygosity with another DDX11 variant in a child with cochlear nerve agenesis [13] and in the homozygous state in the Chilean patient described by Leal et al. [15]. The markedly different cochlear and cochlear-nerve phenotypes among individuals carrying the same frameshift allele do not support a simple genotype-imaging correlation and are consistent with the absence of an established clinically useful genotype–phenotype correlation for DDX11-related cohesinopathy [1].

From a rehabilitative perspective, the postoperative findings should be interpreted in the context of the child’s underlying neurodevelopmental disorder. Serial LittlEARS measurements provided standardized longitudinal evidence of progressive auditory skill acquisition: the score increased from 0/35 at activation to 10/35 at 8 months, 21/35 at 20 months, and 33/35 at 24 months of auditory age. The Greek-language version was included in the multilingual validation of the LittlEARS, supporting its use for longitudinal assessment of early auditory behavior. At approximately 30 months of auditory age, the CELF, Derbyshire, and STASS assessments provided complementary descriptive information on receptive and expressive language. Because these instruments were not standardized for the Greek pediatric population in the form used, normative scores and language-age equivalents were deliberately not derived. At 33 months of auditory age, PEACH-GR demonstrated functional auditory performance scores of 90% in quiet, 80% in noise, and 85% overall. In contrast, Greek-standardized language assessment demonstrated persistent difficulties relative to chronological-age expectations, with performance at the 10th percentile for descriptive adequacy and word finding and below the 10th percentile for grammatical adequacy. The observed gains therefore document functional progress but should not be interpreted as age-appropriate language development. More broadly, children with hearing loss and additional developmental disabilities may achieve clinically meaningful improvements in auditory awareness and functional communication [31] even when speech and language development remains slower or less complete than in children without additional disabilities [32].

When behavioral outcome assessment is constrained by age or neurodevelopmental status, additional objective auditory evoked-potential measures may be useful. Electrically evoked auditory brainstem responses (eABR, also termed EBERA in some settings), obtained through a cochlear implant or a dedicated electrical stimulation system (e.g., PromStim or ANTS), can provide functional information about auditory-nerve and brainstem pathway responsiveness and may provide complementary functional and prognostic information in selected pediatric implant candidates [33]. Electrically evoked cortical auditory evoked potentials (eCAEPs; related to eALR/ECERA terminology) can provide complementary information about cortical detection of electrical auditory input, including in children with cochlear nerve deficiency [34]. In the present patient, formal postoperative eABR or eCAEP testing was not performed; however, intraoperative electrically evoked neural responses were obtained bilaterally, providing objective confirmation of neural responsiveness at the time of implantation.

The molecular data also require qualification. Whole-exome sequencing identified the pathogenic DDX11 c.1403dup variant in apparent homozygosity, and copy-number analysis did not identify a deletion involving part or all of DDX11. The diagnostic assay was based exclusively on next-generation sequencing, without Sanger confirmation or other orthogonal validation. The molecular findings provided a unifying explanation for the combination of microcephaly, growth restriction, developmental delay, and sensorineural hearing loss and supported disorder-specific counseling and multidisciplinary surveillance. Nevertheless, parental segregation testing was not completed. Thus, although the variant was called homozygous by whole-exome sequencing, segregation and parental carrier status remain unconfirmed.

The main limitation of this report is its single-patient design. Furthermore, the focused literature review may have been influenced by incomplete reporting, overlapping publications, and under-identification of cochlear implant recipients in studies primarily focused on the genetic phenotype. These factors should be considered when interpreting the findings and the available literature on cochlear implantation in WABS.

## 4. Conclusions

Severe-to-profound sensorineural hearing loss in WABS may occur despite normal cochlear morphology and bilaterally visualized cochlear nerves. The present case therefore extends the recognized otologic phenotype beyond the structural inner-ear and neural abnormalities described in many previously reported patients. In this child, simultaneous bilateral cochlear implantation was technically uncomplicated. During subsequent bilateral device use, programming, rehabilitation, maturation, and developmental follow-up, progressive auditory development and functional communication gains were documented. Normal radiological anatomy should not be interpreted as evidence of preserved cochlear function, and implant candidacy should be determined by integrating objective audiological findings, detailed temporal-bone imaging, cochlear nerve assessment, developmental status, molecular diagnosis, and realistic family-centered counseling. Further cases with standardized longitudinal outcomes are needed before broader conclusions regarding prognosis or optimal implantation strategy can be drawn.

## Figures and Tables

**Figure 1:**
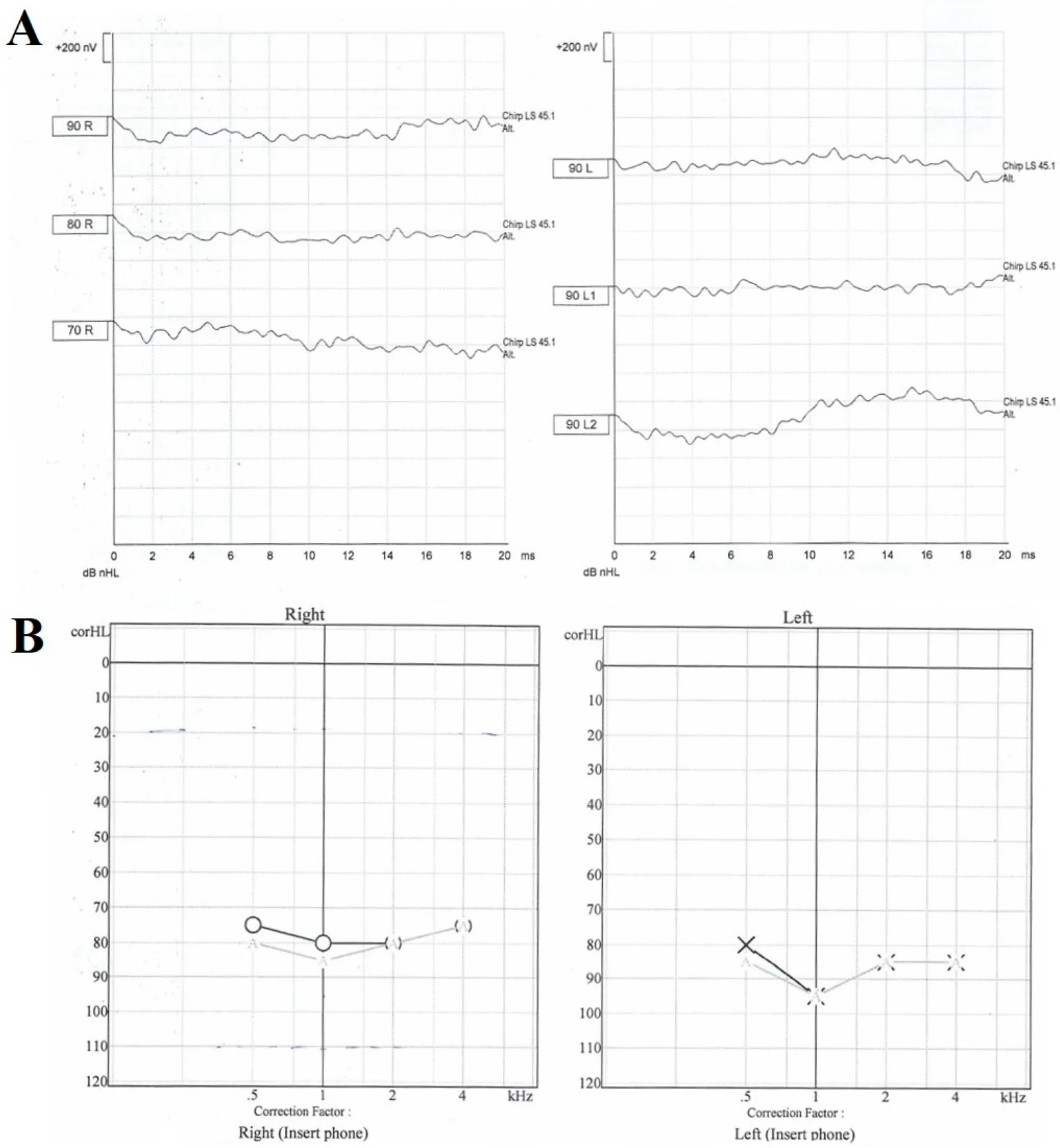
Objective audiological evaluation at 2.5 years of age. (**A**) Auditory brainstem response (ABR): no reproducible waveforms were obtained in either ear at the maximum tested level of 90 dB nHL. (**B**) Auditory steady-state response (ASSR): estimated thresholds were in the severe-to-profound range bilaterally across 0.5, 1, 2, and 4 kHz. Diagnostic ABR and ASSR recordings were obtained using the Interacoustics Eclipse platform with insert earphones.

**Figure 2:**
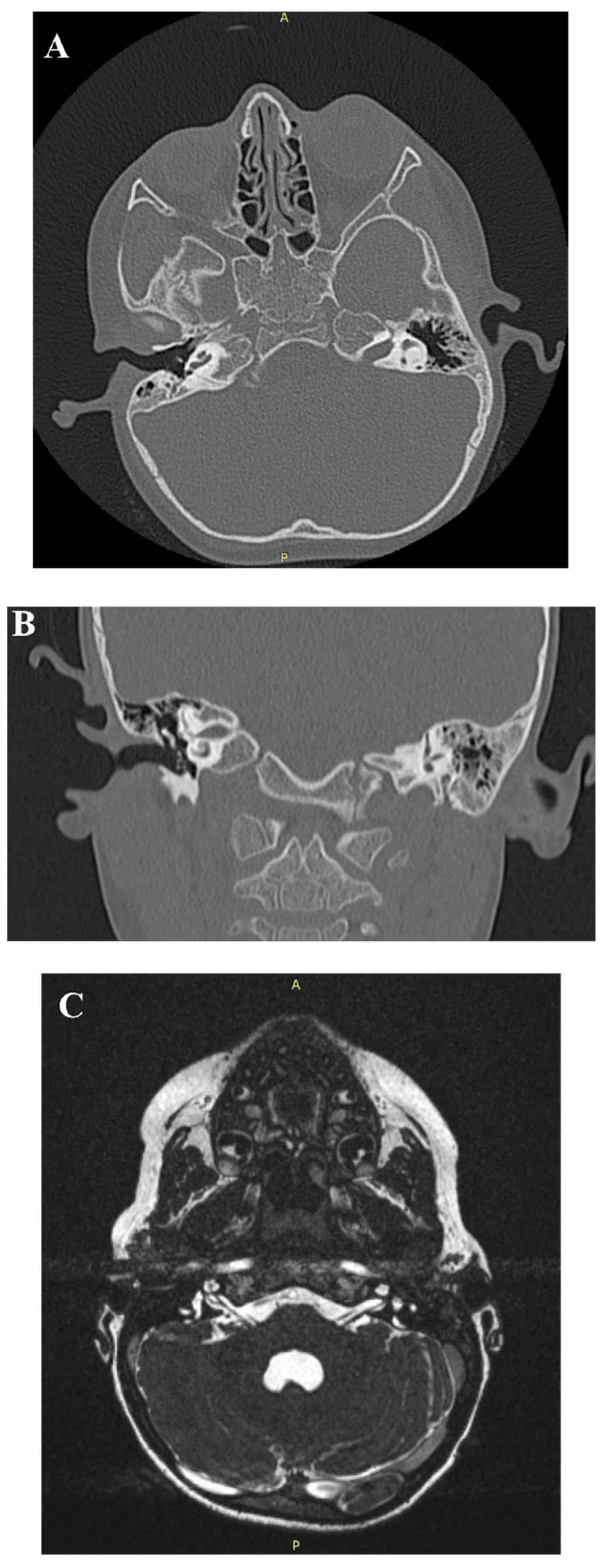
Preoperative temporal-bone imaging. (**A**) Axial high-resolution computed tomography (CT) demonstrates preserved bilateral cochlear and labyrinthine morphology without evidence of congenital inner-ear malformation. (**B**) Coronal high-resolution CT provides complementary visualization of the cochleae and preserved modiolar architecture. Review of the complete CT dataset confirmed 2.5 cochlear turns bilaterally, normally formed round and oval windows, absence of labyrinthitis ossificans, and normal-caliber internal auditory canals. (**C**) Axial high-resolution T2-weighted magnetic resonance imaging (MRI) demonstrates the bilateral internal auditory canals and vestibulocochlear regions. Review of the complete MRI dataset confirmed bilateral cochlear nerves, symmetrically visualized and of normal diameter, without evidence of aplasia or marked hypoplasia.

**Table 1:** Clinical timeline and longitudinal auditory and language outcomes.

Chronological Age/Time Point	Auditory Age	Key Assessment/Outcome
Birth	-	Term birth following an uncomplicated pregnancy; newborn hearing screening was not performed.
2.5 years	Pre-implant	Referral for absent spoken-language development and limited auditory responsiveness. After treatment of bilateral middle-ear effusions, TEOAEs were absent bilaterally; no CM was detected; ABR showed no reproducible response at 90 dB nHL, and ASSR estimates were in the severe-to-profound range.
2.5–3 years	Pre-implant	CT and MRI showed normal cochlear morphology and bilaterally visualized cochlear nerves. Whole-exome sequencing identified a pathogenic DDX11 frameshift variant in apparent homozygosity, supporting the molecular diagnosis of WABS.
3 years	Pre-activation	Simultaneous bilateral cochlear implantation with full electrode-array insertion and satisfactory intraoperative telemetry; no surgical complications.
3 years + 1 month	0 months	Bilateral device activation; LittlEARS 0/35. Intensive auditory–verbal rehabilitation and regular device programming.
3 years + 9 months	8 months	LittlEARS 10/35.
4 years + 9 months	20 months	LittlEARS 21/35.
5 years + 1 month	24 months	LittlEARS 33/35, documenting marked acquisition of early auditory skills.
5 years + 6 months	~30 months	Structured language assessment with CELF, Derbyshire screening, and STASS was interpreted descriptively because Greek normative data were unavailable. The child followed simple verbal commands without visual cues and produced spontaneous short phrases.
5 years + 10 months	33 months	PEACH-GR 90% in quiet, 80% in noise, 85% overall; Action Picture Test descriptive adequacy 10th percentile, grammatical adequacy <10th percentile; Word Finding Vocabulary Test 10th percentile. Percentiles based on chronological-age norms; auditory-age normative comparison not possible because auditory age was below the 4-year lower limit of the tests.

**Table 2:** Published patient-level cochlear implantation reports in Warsaw Breakage Syndrome.

Report	Hearing Phenotype	Temporal-Bone/Cochlear Nerve Findings	Cochlear Implantation	Reported Outcome
Rabin et al., 2019 [14], Patient 1	Severe bilateral SNHL diagnosed at 1 year	Childhood MRI reportedly abnormal, but report unavailable; cochlear anatomy not described	Right unilateral CI at 2 years	Slow speech development; at 21 years, the patient communicated needs, although speech remained unclear
Rabin et al., 2019 [14], Patient 2	Profound bilateral SNHL diagnosed at 6 months	Bilateral cochlear hypoplasia and additional structural brain abnormalities	Bilateral CI at 2 years; whether simultaneous or sequential was not reported	Progressive communication gains; at 16 years, the patient combined words and communicated most needs, although speech remained unclear
Bottega et al., 2019 [11]; Brotto et al., 2020 [12]	Congenital profound bilateral SNHL in the younger of two sisters	Bilateral incomplete partition type II without enlarged vestibular aqueducts	Sequential bilateral CI: right at 1.5 years; left at 4 years	Aided PTA 32.5 dB nHL right and 50 dB nHL left; auditory attention, language production, and communication improved after remapping and second-CI use
Leal et al., 2024 [15]	Abnormal bilateral neonatal ABRs; later described as left deafness and severe right hearing loss	Temporal-bone and cochlear-nerve findings not reported	Left CI at corrected age 21 months	Device-specific postoperative aided thresholds and speech-perception outcomes were not reported; implant benefit could not be assessed reliably
Molnar et al., 2026 [16]	Bilateral severe SNHL	Bilateral type I cochlear hypoplasia; absent modiolus; cyst-like fusion of middle and apical turns	Left unilateral CI at ~2.9 years of age after 3D reconstruction and individualized array selection	Appropriate electrode placement, improved auditory thresholds, and early rehabilitation benefit
Present case	Bilateral severe-to-profound SNHL identified at 2.5 years	Normal cochlear morphology; both cochlear nerves visualized without aplasia or marked hypoplasia	Simultaneous bilateral CI at 3 years	LittlEARS 0/35 at activation, 10/35 at 8 months, 21/35 at 20 months, and 33/35 at 24 months; by ~30 months auditory age, simple verbal-command comprehension without visual cues and spontaneous short phrases. At 33 months of auditory age, PEACH-GR scores were 90% in quiet, 80% in noise, and 85% overall. On Greek-standardized language assessment, performance corresponded to the 10th percentile for descriptive adequacy, below the 10th percentile for grammatical adequacy, and the 10th percentile on the Word Finding Vocabulary Test, based on chronological-age norms

Abbreviations: ABR, Auditory Brainstem Response; CI, cochlear implant; PTA, pure-tone average; SNHL, sensorineural hearing loss. The table includes publications identified through the focused PubMed/MEDLINE search and reference-list screening that provided identifiable patient-level information on cochlear implantation. It is not intended to represent an exhaustive catalogue of all individuals with WABS. The reports by Bottega et al. and Brotto et al. describe the same implanted patient.

## Data Availability

All data supporting the findings of this study are contained within this article.

## Abbreviations

ABR	Auditory Brainstem Response
ASSR	Auditory Steady-State Response
CELF	Clinical Evaluation of Language Fundamentals
CI	Cochlear Implant
CM	Cochlear Microphonic
CT	Computed Tomography
eABR	Electrically Evoked Auditory Brainstem Response
eCAEP	Electrically Evoked Cortical Auditory Evoked Potential
LEAQ	LittlEARS Auditory Questionnaire
MRI	Magnetic Resonance Imaging
OWL	Outer-Wall Length
PEACH-GR	Greek version of the Parents’ Evaluation of Aural/Oral Performance of Children
PTA	Pure-Tone Average
SNHL	Sensorineural Hearing Loss
STASS	South Tyneside Assessment of Syntactic Structures
TEOAE	Transient-Evoked Otoacoustic Emission
WABS	Warsaw Breakage Syndrome
